# Treatment intensification and therapeutic inertia of antihypertensive therapy among patients with type 2 diabetes and hypertension with uncontrolled blood pressure

**DOI:** 10.1038/s41598-024-63617-4

**Published:** 2024-06-01

**Authors:** Kim Sui Wan, Foong Ming Moy, Muhammad Fadhli Mohd Yusoff, Feisul Mustapha, Mastura Ismail, Halizah Mat Rifin, Kishwen Kanna Yoga Ratnam, Hasimah Ismail, Kah Kian Chong, Noor Ani Ahmad, Noran Naqiah Hairi

**Affiliations:** 1 Institute for Public Health, National Institutes of Health, Ministry of Health Malaysia, Setia Alam, 40170 Shah Alam, Selangor, Malaysia; 2 Centre for Epidemiology and Evidence-Based Practice, Department of Social and Preventive Medicine, Faculty of Medicine, Universiti Malaya, 50603 Federal Territory of Kuala Lumpur, Malaysia; 3 Disease Control Division, Federal Government Administration Centre, Ministry of Health, 62590 Putrajaya, Malaysia; 4 Perak State Health Department, Ministry of Health Malaysia, 30000 Ipoh, Perak, Malaysia; 5 Family Health Development Division, Federal Government Administration Centre, Ministry of Health Malaysia, 62590 Putrajaya, Malaysia; 6 Medical Department, Hospital Port Dickson, Negeri Sembilan, 71050 Port Dickson, Malaysia; 7 Faculty of Public Health, Universitas Airlangga, Surabaya City, 60115 East Java Indonesia

**Keywords:** Diabetes, Type 2 diabetes

## Abstract

Treatment intensification is essential to ensure guideline targets are attained in diabetes patients. The failure to intensify treatment when the targets are not achieved is therapeutic inertia. This study aimed to determine the proportions and factors associated with treatment intensification and therapeutic inertia of antihypertensive therapy in type 2 diabetes patients with uncontrolled hypertension in Malaysia. A retrospective cohort analysis was conducted utilising registry data. Diabetes hypertensive patients with uncontrolled baseline systolic or diastolic blood pressure were included. Treatment intensification was the increase in the number of antihypertensive agents from the index treatment. Therapeutic inertia was the absence of treatment intensification when the second blood pressure reading was still uncontrolled. About 6956 patients were followed up over 2.5 ± 1.1 person-years. Treatment intensification was observed in 29.8% of patients, while 38.6% had therapeutic inertia. Chinese, Indian, and ‘others’ ethnic groups, retinopathy, more antihypertensive agents, and higher systolic blood pressure were associated with therapeutic inertia. Underweight, overweight patients and those with dyslipidaemia had lower risks for therapeutic inertia. The results indicate suboptimal quality of care in public health clinics in Malaysia. Further studies are needed to determine the underlying causes to formulate precise interventions to tackle the problem in Malaysia.

## Introduction

Diabetes and hypertension are two of the most significant global public health concerns with enormous socioeconomic ramifications^1,2^. The global disease burden of diabetes increased steadily from 1990 to 2017 and is predicted to continue rising from 2018 to 2025 in terms of incidence, prevalence, death, and disability-adjusted life-years (DALYs)^1^. Meanwhile, the worldwide number of people aged 30–79 with hypertension has doubled from 1990 to 2019^2^. The proportion of people with blood pressure (BP) below 140/90 mmHg in 2019 was only 23% for women and 18% for men^2^. 

Malaysia is a multi-ethnic country in Asia, and diabetes and hypertension are highly prevalent in this upper-middle-income nation^3^. The prevalence of diabetes and hypertension among the general adult population was 18.3% and 30.0%, respectively^3^. Among people with type 2 diabetes mellitus (T2DM), over 80% have hypertension comorbidity^4,5^, and most of them receive treatments at public healthcare facilities, which are heavily subsidised by general taxation^3^.

Clinical practice guidelines recommend the achievement of glycosylated haemoglobin A1c (HbA1c), lipid, and BP targets to prevent diabetes complications^6–8^. Among these parameters, BP is often the most challenging target to achieve, as reported in Europe, Canada, China, and Malaysia^4,9–11^. One main reason for suboptimal risk factor control in clinical practice is the lack of treatment intensification, an established indicator of therapeutic inertia^12^. Therapeutic inertia is the lack of adjustment to a therapeutic regimen when the treatment targets are not attained in diabetes patients^13^. According to a systematic review, among patients with at least one HbA1c reading above a cut-off, determining the proportion of those who had treatment intensification within a period of time is the most commonly used method to quantify therapeutic inertia^14^. Some studies had a few follow-ups with more than one HbA1c reading used in quantifying therapeutic inertia^14^.

Therapeutic inertia in managing hypertension, diabetes, and dyslipidaemia may account for up to 80% of strokes and heart attacks^15^. We have previously reported treatment intensification of antidiabetic therapy among T2DM patients in Malaysia^16^. However, new evidence shows the legacy advantages of controlling multiple cardiovascular risk factors. The legacy effect is the prolonged benefits of early blood glucose, BP, and lipid control for preventing cardiovascular complications in diabetes patients^17^. A two-decade follow-up study to a landmark randomised control trial reported decreased all-cause mortality, macrovascular complications, and microvascular outcomes in the intensively treated arm compared to the control group^18^.

Therefore, there is a strong need to go beyond the glycaemic-centred view of therapeutic inertia in diabetes^12^. Besides that, limited local studies are exploring treatment intensification and therapeutic inertia in BP management, posing critical knowledge gaps in a country with very high diabetes and hypertension prevalence. Thus, we aim to determine the proportion and factors associated with the treatment intensification and therapeutic inertia of antihypertensive therapy in T2DM patients with uncontrolled hypertension in Malaysia.

## Methods

### Study design and source of data

We conducted secondary data analysis of a five-year retrospective open cohort of T2DM patients under the care of all public health clinics in Negeri Sembilan state, Malaysia, and the particulars had been previously published^4,19,20^. Negeri Sembilan is one of the sixteen states and federal territories in Malaysia and is located south of the capital city, Kuala Lumpur. The state was chosen due to its high prevalence of diabetes in Malaysia, potentially better data quality, and similar demographic characteristics and degree of urbanisation as the national average^4,5,19^. In an open cohort, patients could leave or be added over the study period, and this dynamic membership mirrored the real-world scenario^21^. This patient cohort benefitted from a large real-life clinical dataset and was representative of the national average^4,5^. Thus, the research results could potentially be externally generalised to T2DM patients seeking treatments at public health clinics in Malaysia^4^.

This cohort dataset was assembled by merging six datasets from the National Diabetes Registry for Negeri Sembilan state^20^.The registry monitored cardiovascular risk factors control and complications in diabetes patients who received care from facilities under the Ministry of Health^5^. This database contained two types of datasets: the registry and annual clinical audit datasets. The former had basic demographic and follow-up information on patients with T2DM, type 1 diabetes mellitus, and other diabetes types in hospital and public health clinic settings^5^. Meanwhile, the clinical audit datasets were created annually when about a subset of T2DM patients in public clinics were randomly sampled for audits^5^. The clinical audit datasets contained clinical and medication information and laboratory investigation results. Even though patients usually had a few clinic follow-ups each year, only the last observed clinical information was captured to represent the entire year’s performance^5^.

The registry is essential for the Ministry of Health to monitor diabetes care quality in Malaysia^5^. However, the cross-sectional nature of the annual clinical audits disallows longitudinal data analysis. Data integration can solve the issue by assembling data from separate datasets with same variables, thus creating a novel dataset that gives better flexibility in the analysis compared to the independent analysis of individual datasets^22^. Clinical audit datasets from the year 2013 to 2017 were combined after matching identical identifiers: national registration identity card numbers and names. Only patients with at least two clinical readings were included in the cohort dataset, constituting a follow-up. These patients were followed up per usual clinical practice in real-world settings with no extra procedures in this observational retrospective secondary data analytical study. Next, follow-up and smoking information were taken from the registry dataset to create the final cohort dataset. Some study findings of this population-based open cohort dataset have been published^4,16,19,20^.

**Figure 1:**
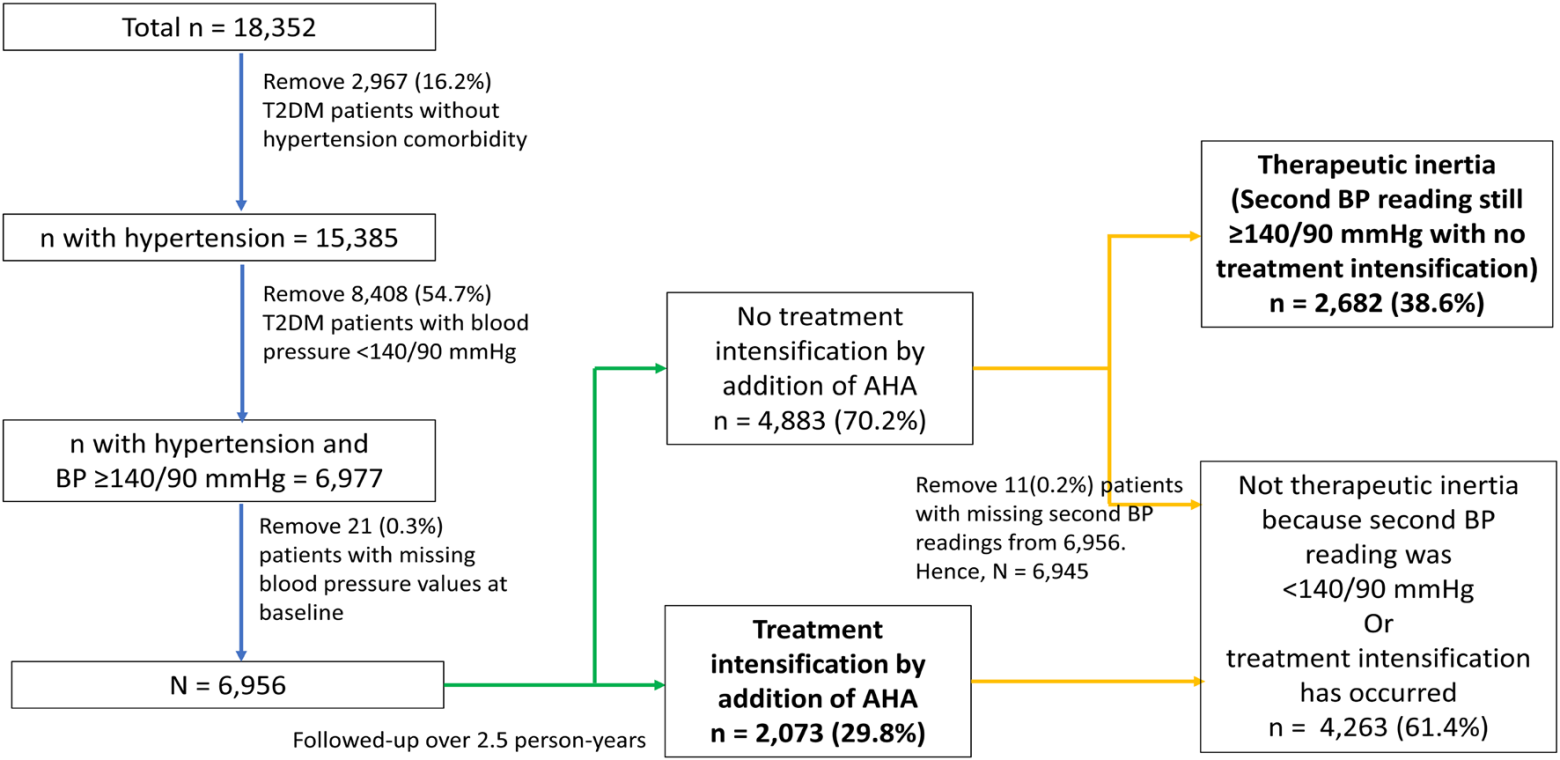
Flow diagram showing the selection of patients, definitions, and results.

The original number of patients in the cohort dataset was 18,352, as shown in Fig. 1. The total number of patients in the Negeri Sembilan registry dataset as of 1 October 2017, when the datasets were retrieved, was 94,023. Hence, the cohort dataset represented 19.5% (18,352 divided by 94,023) of the overall treated population. It is important to stress that the cohort dataset was assembled from annual clinical audit datasets, whereby patients were randomly selected every year and independent of whether patients had previously been selected for audits. Thus, patients in the cohort dataset were truly random and representative of the overall population.

A total of 90,897 independent clinical audit data were available from the years 2013 to 2017: 16,365 for 2013, 16,546 for 2014, 17,179 for 2015, 19,535 for 2016, and 21,272 for 2017. Only patients with exact identifiers of national registration identity card numbers and names (in other words, duplicates) were included in the cohort dataset. As the number of patients in the cohort dataset was 18,352, the chance of patient duplication over the five years of datasets was 20.2% or one in every five clinical audits (18,352 divided by 90,987).

### Eligibility criteria

For this analysis, the inclusion criteria were: (a) T2DM patients aged ≥ 18 years with hypertension comorbidity; (b) uncontrolled systolic BP ≥ 140 mmHg or diastolic BP ≥ 90 mmHg at the study baseline. These particular BP cut-offs were adopted from the American Diabetes Association clinical guidelines for primary care providers^6^. They were used because the guidelines were widely used and represented the clinical values used to initiate treatment in patients with hypertension^23^, which could be used to compare with similar studies. From the original dataset with 18,352 patients, a total of 11,375 were not hypertensive or had BP < 140/90 mmHg. After excluding another 21 patients with missing baseline BP values, the number of eligible samples was 6956, as shown in Fig. 1.

### Dependent variables

Treatment intensification was defined as an increase in the number of antihypertensive agents (AHA) from the baseline index treatment^14^. The number of AHA was the sum of angiotensin-converting enzyme inhibitors (ACE-I), angiotensin-receptor blockers, peripheral alpha-blockers, calcium channel blockers, central-acting agents diuretics, beta-blockers, and diuretics. The index treatment was categorised as zero, one, two, and ≥ three AHA. The percentage of treatment intensification was calculated from the number of patients with treatment intensification divided by the number of eligible samples (n = 6956), as seen in Fig. 1. If a patient’s medications were intensified several times over the study period, this would be considered a single event of treatment intensification for this particular patient. This was because the denominator for the treatment intensification percentage was the number of eligible patients, not the number of clinical encounters.

Therapeutic inertia was the failure to intensify treatment, although the second BP readings were still uncontrolled with systolic BP ≥ 140 mmHg or diastolic BP ≥ 90 mmHg. The percentage of therapeutic inertia was calculated from the number of patients with therapeutic inertia divided by the number of eligible samples after removing those with missing second BP readings (n = 6945), as seen in Fig. 1. Essentially, therapeutic inertia was the reverse of treatment intensification, but it further accounted for the second uncontrolled BP readings to highlight ‘inappropriate inaction.’ Patients without treatment intensification with second BP readings within treatment targets (< 140/90 mmHg) did not have therapeutic inertia and were considered ‘appropriate inaction.’ By clearly defining treatment intensification and therapeutic inertia, we could differentiate inappropriate and appropriate inaction and yield more accurate results.

### Independent variables

The baseline characteristics were age, sex, ethnic groups, duration of diabetes, comorbidities (dyslipidaemia, overweight, obesity), diabetes complications (stroke, ischemic heart disease, nephropathy, retinopathy, and foot-related complications), treatments (modes of diabetes treatment, AHA, lipid-lowering agents, and antiplatelet agents) and metabolic control (HbA1c, BP, and LDL-cholesterol). The stages of hypertension were categorised as mild (systolic BP of 140–159 and/or diastolic BP of 90–99 mmHg), moderate (systolic BP of 160–179 and/or diastolic BP of 100–109 mmHg), and severe (systolic BP of ≥ 180 and/or diastolic BP of ≥ 110 mmHg). The clinical practice guidelines generally recommended HbA1c < 7.0% and LDL < 2.6 mmol/L for T2DM patients^6–8^.

### Statistical analysis

The analyses were done utilising the IBM SPSS Statistics 23. Continuous variables were reported as mean ± standard deviation for normally distributed data, whereas median (interquartile range) was used for skewed data. Frequencies and percentages were presented for categorical variables. 95% confidence intervals were reported for patients with treatment intensification and therapeutic inertia. For bivariate analysis, Pearson chi-square tests were conducted to compare the proportions. Meanwhile, Student *t*-tests were used to compare the means, while Mann–Whitney tests were carried out to compare the medians.

As the dataset was an open cohort and the exact dates for treatment intensification were unknown, the data captured was interval-censored; in other words, the event was known to have happened within a specific interval but without knowing the exact time^24^. Thus, we conducted complementary log–log transformations to get proportional hazard models to account for interval censoring^24^. Wald chi-square and maximum likelihood estimation were utilised to fit multivariate proportional hazards models to adjust for potential confounders in the study. For the selection of the final models, we assessed the model fit using Akaike’s information criterion (AIC), Bayesian information criterion (BIC), and log-likelihood. The information criteria of these goodness-of-fit tests were in the smaller-is-better form, as generated by the SPSS software. We also reported the Omnibus test, which compared the fitted model against the baseline model. A *P*-value of < 0.05 was considered statistically significant. The hazard ratios and 95% confidence intervals were reported.

We conducted sensitivity analyses using higher baseline BP cut-offs (≥ 150/95, ≥ 160/100, ≥ 170/105, and ≥ 180/110 mmHg) as inclusion criteria to determine if different BP levels would affect the proportions of patients with treatment intensification and therapeutic inertia. We used the same higher BP cut-offs for the second BP readings. For example, when the baseline BP was ≥ 150/95 mmHg, the second BP reading was also ≥ 150/95 mmHg. In addition, we also included the cut-off of ≥ 140/80 mmHg, the recommended BP target by the Malaysian Clinical Practice Guidelines on T2DM management^7^. Such information would be useful for the local settings.

### Ethics

The Medical Research and Ethics Committee of the Ministry of Health Malaysia approved this study (NMRR-1-2731-44032) and waived the need for written informed consent because the data was analysed retrospectively with no patient identification information. All methods followed the Malaysian Good Clinical Practice Guidelines and the Declaration of Helsinki.

## Results

A total of 6956 eligible patients were followed up over a mean of 2.5 ± 1.1 person-years, and 29.8% (95% CI 28.7–30.9) had treatment intensification (Fig. 1). When the second BP reading was accounted for, 38.6% (95% CI 37.5–39.8) of patients had therapeutic inertia. Appropriate inaction was observed in 2192 (31.6%) of patients.

For the baseline characteristics, 61.6% were females, 65.9% were Malays, and 54.1% were older patients aged ≥ 60 (Table 1). The median duration of diabetes was 5.0 (7.0) years, and 5.4% of patients were smokers. About 74.0% were overweight/obese, and 80.4% had dyslipidaemia. Stroke, ischemic heart disease, retinopathy, nephropathy, and foot complications were observed in 1.2%, 3.5%, 3.2%, 6.7%, and 0.9% of them, respectively. The oral hypoglycaemic agent was the most common treatment mode used in 90.6% of patients. More patients (36.6%) were treated with two AHA, and 74.2% and 33.5% were on lipid-lowering and antiplatelet agents, respectively. The mean clinical target values were 7.9% for HbA1c, 150.6 mmHg for systolic BP, 82.9 mmHg for diastolic BP, and 2.97 mmol/L for LDL-cholesterol.

**Table 1:** Baseline characteristics of patients, n = 6956.

Characteristics	n (%)
Age, mean ± SD, years	60.6 ± 10.1
< 60 years	3192 (45.9)
≥ 60 years	3764 (54.1)
Sex	
Male	2668 (38.4)
Female	4288 (61.6)
Ethnicity	
Malay	4582 (65.9)
Chinese	1238 (17.8)
Indian	1110 (16.0)
Others	26 (0.3)
Diabetes duration, median (IQR), years	5.0 (7.0)
Less than five years	3049 (43.8)
Five to ten years	2553 (36.7)
More than ten years	1354 (19.5)
Body mass index (n = 6630)	
Mean ± SD, kg/m^2^	28.3 ± 5.1
Underweight, < 18.5 kg/m^2^	64 (1.0)
Normal, 18.5–24.9 kg/m^2^	1661 (25.0)
Overweight, 25.0–29.9 kg/m^2^	2658 (40.1)
Obese, ≥ 30.0 kg/m^2^	2247 (33.9)
Smoking	376 (5.4)
Dyslipidaemia	5595 (80.4)
Stroke	80 (1.2)
Ischemic heart disease	242 (3.5)
Retinopathy	223 (3.2)
Nephropathy	466 (6.7)
Foot complication	65 (0.9)
Diabetes treatment modality	
Lifestyle modification only	178 (2.5)
Oral hypoglycaemic agent (OHA) only	4764 (68.5)
Insulin only	479 (6.9)
Both OHA and insulin	1535 (22.1)
Number of antihypertensive agents (AHA)	
Zero	157 (2.3)
One	2030 (29.2)
Two	2547 (36.6)
≥ Three	2222 (31.9)
Lipid-lowering agents	5162 (74.2)
Antiplatelet agents	2328 (33.5)
HbA1C, % mean ± SD, n = 6,950	7.90 ± 2.00
Systolic BP, mmHg mean ± SD	150.6 ± 13.2
Diastolic BP, mmHg mean ± SD	82.9 ± 9.9
LDL-C, mmol/L mean ± SD, n = 6,945	2.97 ± 0.98

*AHA* antihypertensive agents, *BP* blood pressure, *HbA1c* glycosylated haemoglobin A1c, *LDL-C* low-density lipoprotein cholesterol, *OHA* oral hypoglycaemic agent.

Treatment intensification was more commonly observed in the Malay ethnic group and underweight patients (Supplementary Table S1). In contrast, patients with dyslipidaemia, nephropathy, lipid-lowering agents, and a higher number of AHA had lower treatment intensification. Patients with treatment intensification had significantly higher HbA1c, systolic BP, diastolic BP, and LDL-cholesterol. Generally, as the systolic and diastolic BP classes increased, the proportion of treatment intensification increased correspondingly.

Table 2 shows the factors associated with treatment intensification of antihypertensive therapy. Compared to the Malay ethnic group, treatment intensification was less likely in Chinese and Indian ethnicities. As the number of AHA increased, the hazard ratios also decreased correspondingly. Patients with three or more AHA were ten times less likely to have treatment intensification than those without AHA. This was the strongest factor in the model, as evidenced by the highest Wald chi-square value. In contrast, underweight, overweight, and obese patients were more likely to have treatment intensification than normal BMI patients. Users of antiplatelet agents and patients with uncontrolled HbA1c ≥ 7.0% were also more likely to have treatment intensification. As the systolic BP classes increased, the adjusted hazard ratios increased accordingly. Patients with systolic BP of ≥ 180 mmHg were 2.9 times more likely to have treatment intensification than those with systolic BP of < 140 mmHg.

**Table 2:** Factors associated with treatment intensification, n = 6624.

Characteristics	Wald Chi-squares	Adjusted hazard ratios	95% confidence intervals	*P* values
Ethnicity				
Malay		1.00		
Chinese	7.23	0.84	0.74–0.95	0.007
Indian	7.39	0.84	0.74–0.95	0.007
Others	0.14	0.86	0.38–1.92	0.706
Body mass index				
Underweight	4.78	1.59	1.05–2.40	0.029
Normal		1.00		
Overweight	13.28	1.24	1.11–1.40	< 0.001
Obese	8.27	1.20	1.06–1.36	0.004
Number of antihypertensive agents				
Zero		1.00		
One	59.71	0.42	0.34–0.53	< 0.001
Two	141.35	0.26	0.21–0.33	< 0.001
≥ Three	362.88	0.10	0.08–0.12	< 0.001
Use of antiplatelet agents	7.45	1.14	1.04–1.26	0.006
Systolic blood pressure				
< 140 mmHg		1.00		
140–159 mmHg	14.53	1.47	1.21–1.79	< 0.001
160–179 mmHg	34.91	1.93	1.55–2.40	< 0.001
≥ 180 mmHg	52.24	2.86	2.15–3.81	< 0.001
HbA1C ≥ 7.0%	7.12	1.14	1.03–1.25	0.008

Akaike’s information criterion (AIC): 1340.1, Bayesian information criterion (BIC): 1442.1, and log likelihood: − 655.1. The information criteria of these goodness-of-fit tests were in the smaller-is-better form and were deemed the best fitted model. The model fit improved compared to the baseline model because the Omnibus test was significant, P < 0.001.

Therapeutic inertia was commonly observed in the Chinese ethnic group, normal bodyweight patients, those with retinopathy, using ≥ three AHA, and those with higher systolic BP (Supplementary Table S1). In the multivariate model, Chinese, Indian, and ‘others’ ethnic groups, retinopathy, increasing number of AHA, and higher systolic BP were associated with high adjusted hazard ratios for therapeutic inertia. Patients with ≥ 3 AHA were six times as likely to have therapeutic inertia, and this was the strongest factor in the model. Meanwhile, underweight, overweight patients and patients with dyslipidaemia had lower risks for therapeutic inertia (Table 3).

**Table 3:** Factors associated with therapeutic inertia, n = 6621.

Characteristics	Wald Chi-squares	Adjusted hazard ratios	95% confidence intervals	*P* values
Ethnicity				
Malay		1.00		
Chinese	8.34	1.16	1.05–1.29	0.004
Indian	4.69	1.13	1.01–1.26	0.030
Others	9.58	2.27	1.35–3.81	0.002
Body mass index				
Underweight	4.36	0.60	0.38–0.97	0.037
Normal		1.00		
Overweight	9.34	0.86	0.77–0.95	0.002
Obese	1.62	0.94	0.84–1.04	0.204
Dyslipidaemia	4.32	0.90	0.82–0.99	0.038
Retinopathy	5.59	1.29	1.04–1.59	0.018
Number of antihypertensive agents				
Zero		1.00		
One	14.70	2.58	1.59–4.17	< 0.001
Two	27.02	3.58	2.21–5.78	< 0.001
≥ Three	53.77	6.02	3.72–9.73	< 0.001
Systolic blood pressure, per mmHg	5.14	1.003	1.0004–1.006	0.023

Akaike’s information criterion (AIC): 3858.6, Bayesian information criterion (BIC): 3947.0, and log likelihood: − 1916.3. The information criteria of these goodness-of-fit tests were in the smaller-is-better form and were deemed the best fitted model. The model fit improved compared to the baseline model because the Omnibus test was significant, P < 0.001.

**Figure 2:**
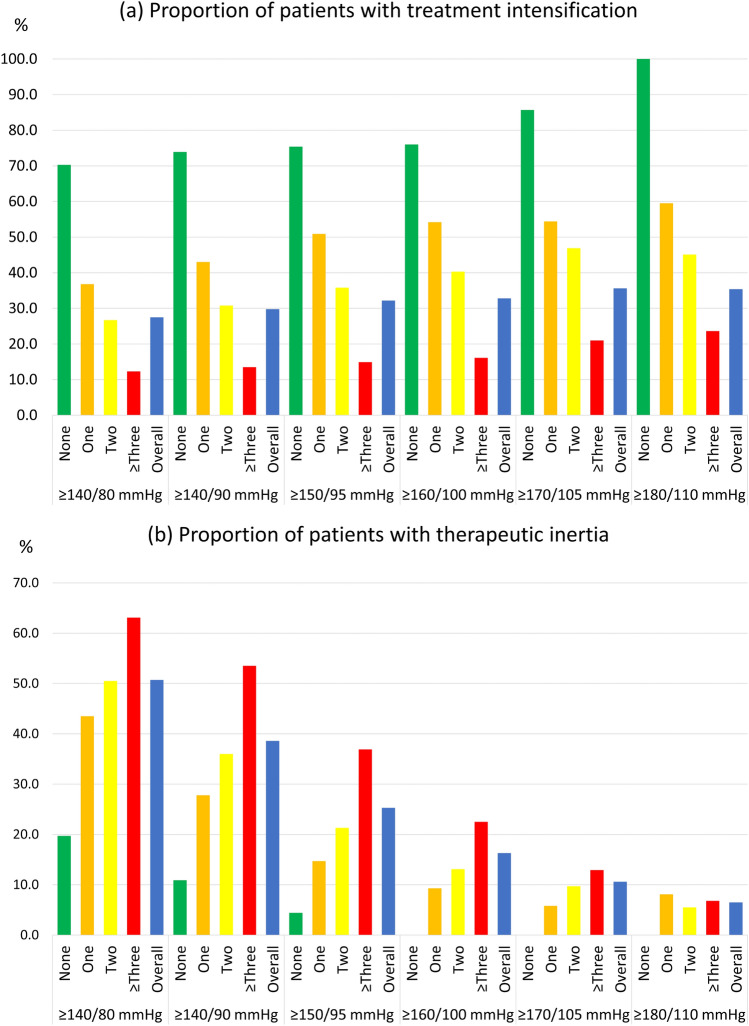
Sensitivity analyses using different index blood pressure and number of antihypertensive agents.

Figure 2 illustrates the sensitivity analyses using different index BP and treatments, while Supplementary Tables S2 and S3 detail the results. Within each BP cut-off, the proportion of patients with treatment intensification consistently decreased as the number of AHA increased (Fig. 2a). When the index BP increased, treatment intensification generally rose across all treatment categories. Meanwhile, the proportion of patients with therapeutic inertia generally increased as the number of AHA increased within each BP cut-off (Fig. 2b). When the index and second BP readings increased, therapeutic inertia generally reduced across all treatment categories.

## Discussion

This study determined the proportion of diabetes hypertensive patients with uncontrolled blood pressure who had treatment intensification and therapeutic inertia. Only three in ten patients had treatment intensification over a mean follow-up of 2.5 person-years. Alarmingly, almost 40% of our patients had therapeutic inertia where there was no treatment intensification, although the BP was still uncontrolled. Their antihypertensive therapies had to be intensified because their BP readings were not meeting treatment targets, as stipulated by clinical practice guidelines^6–8^. Step-wise treatment intensifications by adding AHA from complementary drug classes were necessary to achieve the therapy goal and prevent or delay disease complications^6–8^.

The low proportion of treatment intensification among our patients was unsurprising. In a large population-based cohort study in Italy, 64% of patients on monotherapy failed to receive treatment intensification in the third year of follow-up^25^. In the United States, 83.2% of eligible hypertensive patients did not receive treatment intensification with new medications during primary care visits^26^. A local study in three health clinics reported that between 34 and 65% of patients with uncontrolled BP had therapeutic inertia^27^. In a randomised control study, the prevalence of therapeutic inertia varied between 56 and 60%^28^. A retrospective cohort study using the United Kingdom general practice database found that 33.1% of patients had therapeutic inertia^29^. These study findings were comparable to our results.

Meanwhile, a Dutch study among general practitioners reported that 87% of patients with uncontrolled BP had therapeutic inertia^30^. In Colombia, 81.8% of medical consultations among eligible patients had no treatment modification^31^. The relatively higher prevalence of therapeutic inertia in the Dutch and Colombian studies can be partly explained by their shorter study duration. Studies with longer study duration with several follow-ups tend to have lower proportions of therapeutic inertia^14^. Overall, all these studies demonstrated highly prevalent problems of therapeutic inertia in different regions of the world, making it a challenging public health concern for the foreseeable future^32^.

We observed ethnic differences whereby Chinese and Indian ethnic groups were more likely to have therapeutic inertia than their Malay counterparts. Ethnic differences in therapeutic inertia have been reported in the literature. For example, therapeutic inertia was lower in non-Hispanic Black compared to non-Hispanic White participants in the standard treatment arm of a randomised control study^28^. owever, our results were inconsistent with a Malaysian study involving three public health clinics, whereby no statistical significance was found between the three major ethnicities in Malaysia with therapeutic inertia^27^. Various factors, such as sociodemographic factors, health literacy level, medication adherence, baseline BP, multimorbidity, and access to the healthcare system, may interact with ethnicities, resulting in different findings^12,28,32^. As little published data indicates if therapeutic inertia varies by ethnic groups^32^, we recommend further studies to investigate this issue in our multi-ethnic country.

An interesting study finding was the negative association between underweight and therapeutic inertia. This has clinical significance because evidence is emerging that being underweight may be another independent CVD risk factor in which body mass index has a U-shaped curve relationship^33^. The U- or J-shaped relationship between body mass index and mortality, termed the ‘obesity paradox’, poses a significant challenge to the obesity-disease paradigm (obesity as a risk factor for numerous non-communicable diseases such as CVD, diabetes, hypertension, and dyslipidaemia)^33,34^. Being underweight may be associated with several clinical factors, such as sarcopenia, ageing, poor nutritional status, and poor metabolic control (i.e., metabolically obese underweight)^33^. Hence, it makes clinical sense that therapeutic inertia is less likely to be observed among such patients. Alternatively, the association could be simply due to random errors due to the relatively low number of underweight patients in this study.

We found that overweight and obese patients and those with dyslipidaemia and higher systolic BP were less likely to have therapeutic inertia. These are all risk factors for adverse complications, including mortality, and clinical practice guidelines have recommended specific treatment goals^6–8^. Hence, our observations may reflect better clinical care among these high-risk patients. Studies elsewhere have similarly reported higher systolic BP and near-target systolic BP being independent key factors related to therapeutic inertia^30,35^. Meanwhile, antiplatelet agents could be a proxy indicator for cardiovascular diseases, and better treatment intensification of AHA is expected in this group of patients as the clinical practice guidelines recommended more stringent BP targets for them^6–8^. Patients with retinopathy had a higher risk of therapeutic inertia, and competing interest in managing the higher priority end-organ damage could partly explain it^36^.

We observed that being on more AHA posed challenges to intensifying the treatment regimens in a dose-related manner. In other words, the stepping up of AHA itself faced resistance, and three or more AHA was the strongest associated factor for treatment intensification and therapeutic inertia. Studies among diabetes patients have similarly reported that more oral hypoglycaemic agents increased the likelihood of therapeutic inertia^16,37^. Multidrug treatments, including polypharmacy, pose several clinical challenges, such as therapeutic inertia, poor patient compliance, and treatment complexity (e.g., drug-drug interaction and side effects)^38^.

Recent studies have highlighted the suboptimal control of HbA1c, BP, and LDL-cholesterol among T2DM patients in Malaysia^20,39^. The disconnection between guideline recommendations and actual prescribing practices and the deficiency in clinical care processes threaten the quality of disease management^20,39^. Our current analysis and other emerging evidence strongly suggest therapeutic inertia in diabetes, hypertension, and dyslipidaemia management among T2DM patients in Malaysia^16,20,27,40^. However, therapeutic inertia is inadequately described in the local clinical practice guidelines for diabetes and hypertension management^7,23^. Thus, there is an urgent need to raise awareness and priority about the issue among clinicians, program managers, and health policymakers in Malaysia. For example, the American Diabetes Association developed a three-year initiative to address therapeutic inertia in 2020 and beyond^13^. Improving the understanding of therapeutic inertia and adherence to clinical practice guidelines is vital for clinicians to detect this issue in their daily clinical practice^13^. User-friendly decision support tools may also help to improve step-wise treatment intensification to prevent inertia in the future^13^.

The causes of therapeutic inertia are divided into physician factors, patient factors, and system factors that may interact in complex ways^15^. For instance, some general practitioners prioritised lifestyle interventions first while waiting for subsequent blood pressure measurements^30^. Some patients explicitly refused to change their treatment plans^30^. Therefore, developing interventions to reduce therapeutic inertia may best be multifactorial to optimise their effectiveness^15^. Nevertheless, little is known about the critical drivers of therapeutic inertia in Malaysian clinical settings. Hence, we recommend more research to determine its underlying causes so that precise interventions can be formulated. In addition, new research can also focus on specific activities, skills, or ways to improve the achievement of therapeutic targets among T2DM patients^13^.

There are several limitations to this research. The Malaysian National Diabetes Registry did not have details on drug dosages. Thus, dosage adjustment was not included in our study definitions. However, the findings are valid because the 2.5 person-years of follow-up is sufficiently long to adjust any existing antihypertensive regimens to the maximum dosages; after that, the addition of another AHA is clinically indicated^6,7^. It would be more informative if we could also determine the time until treatment intensification. However, the interval-censored data disallowed us from doing that. Besides that, important information such as medication adherence, health literacy levels, and the number of follow-up visits, all of which can influence treatment intensification, were not studied as these data were not captured in the registry^15^. Such information may also interact with ethnicities as described above, and therefore, more comprehensive studies are recommended to understand the relationships between ethnicity and therapeutic inertia in Malaysia. Lastly, the study was limited by the relatively old assembled cohort dataset, which was unavoidable as the cross-sectional nature of the registry disallows longitudinal data analysis.

The sufficiently large and representative population of T2DM patients in Malaysia is the main strength of this study^4,5^. The analysis using real-world clinical data adds crucial knowledge about therapeutic inertia in BP management among diabetes hypertensive patients, a research topic that is relatively unexplored in Malaysia^23^. The sensitivity analyses further increase this study’s internal validity and the understanding of how differing cut-off values affect treatment intensification and therapeutic inertia. The new information may uncover a neglected issue in diabetes management and serve as a baseline for future research and quality improvement^13^. Besides that, our study definition helped to differentiate therapeutic inertia (inappropriate inaction) from appropriate inaction. If we only focused on treatment intensification without accounting for the second BP values, almost 70% of patients would be incorrectly identified as having therapeutic inertia. By quantifying therapeutic inertia and identifying its associated factors, our results provide essential information for health policymakers, program managers, and clinicians to improve the quality of care in Malaysia.

## Conclusions

In conclusion, only around 30% of T2DM patients with uncontrolled hypertension had treatment intensification in public health clinics in Malaysia. Meanwhile, almost four-in-ten patients had therapeutic inertia. Chinese, Indian, and ‘others’ ethnic groups, retinopathy, increasing number of antihypertensive agents, and higher systolic blood pressure were associated with therapeutic inertia. In contrast, underweight and overweight patients and those with dyslipidaemia had lower risks for therapeutic inertia. The results indicate suboptimal quality of diabetes care in public health clinics in Malaysia. Further studies are needed to determine its underlying causes and formulate precise interventions to tackle the issue in Malaysia.

### Supplementary Information

Supplementary Information 1.

Supplementary Information 2.

Supplementary Information 3.

## Data Availability

The National Diabetes Registry dataset retrieved and analysed in this study is not available publicly due to local ethics regulations, and written permission from the Director General of Health Malaysia is required. Please contact Dr Wan Kim Sui at kimsui@moh.gov.my to request the data of this study.
