# Supplementary material for: Treatment intensification and therapeutic inertia of antihypertensive therapy among patients with type 2 diabetes and hypertension with uncontrolled blood pressure
Source: Sci Rep. 2024 Jun 1;14:12625. doi: 10.1038/s41598-024-63617-4 (PMC11144228; doi:10.1038/s41598-024-63617-4)
Supplement: Supplementary file 1 — Supplementary Information 1. [file 41598_2024_63617_MOESM1_ESM.docx]

**Supplementary Table S1.** Characteristics of patients with treatment intensification and therapeutic inertia

| Characteristics | Treatment intensification | | *P* values | Therapeutic inertia | | *P* values |
| --- | --- | --- | --- | --- | --- | --- |
|  | Yes, n (%) | No, n (%) |  | Yes, n (%) | No, n (%) |  |
|  | 2,073 (29.8) | 4,883 (70.2) |  | 2,682 (38.6) | 4,263 (61.4) |  |
| **Age,** mean ± SD, years  <60 years  ≥60 years | 60.1 ± 9.8  988 (31.0)  1,085 (28.8) | 60.9 ± 10.2  2,204 (69.0)  2,679 (71.2) | 0.003  0.053 | 60.8 ± 10.3  1,228 (38.5)  1,454 (38.7) | 60.5 ± 9.9  1,960 (61.5)  2,303 (61.3) | 0.348  0.877 |
| **Sex**  Male  Female | 776 (29.1)  1,297 (30.2) | 1,892 (70.9)  2,991 (69.8) | 0.303 | 1,055 (39.6)  1,627 (38.0) | 1,607 (60.4)  2,656 (62.0) | 0.171 |
| **Ethnicity**  Malay  Chinese  Indian  Others | 1,429 (31.2)  319 (25.8)  319 (28.7)  6 (23.1) | 3,153 (68.8)  919 (74.2)  791 (71.3)  20 (76.9) | 0.002 | 1,699 (37.1)  528 (42.7)  438 (39.5)  17 (65.4) | 2,875 (62.9)  709 (57.3)  670 (60.5)  9 (34.6) | <0.001 |
| **Diabetes duration,** median (IQR), years  Less than five years  Five to ten years  More than ten years | 5.0 (7.0)  943 (30.9)  760 (29.8)  370 (27.3) | 5.0 (7.0)  2,106 (69.1)  1,793 (70.2)  984 (72.7) | 0.128  0.055 | 5.0 (7.0)  1,153 (37.9)  977 (38.3)  552 (40.9) | 5.0 (7.0)  1,892 (62.1)  1,574 (61.7)  797 (59.1) | 0.057  0.146 |
| **Smoker**  Yes  No | 117 (31.1)  1,956 (29.7) | 259 (68.9)  4,624 (70.3) | 0.566 | 148 (39.4)  2,534 (38.6) | 228 (60.6)  4,035 (61.4) | 0.761 |
| **Body mass index** (n = 6,630)  Mean ± SD, kg/m^2^  Underweight, <18.5 kg/m^2^  Normal, 18.5–24.9 kg/m^2^  Overweight, 25.0–29.9 kg/m^2^  Obese, ≥30.0 kg/m^2^ | 28.4 ± 5.0  25 (39.1)  443 (26.7)  828 (31.2)  667 (29.7) | 28.3 ± 5.2  39 (60.9)  1,218 (73.3)  1,830 (68.8)  1,580 (70.3) | 0.601  0.005 | 28.3 ± 5.1  18 (28.1)  687 (41.4)  974 (36.7)  883 (39.4) | 28.3 ± 5.1  46 (71.9)  974 (58.6)  1,679 (63.3)  1,360 (60.6) | 0.611  0.005 |
| **Dyslipidaemia**  Yes  No | 1,632 (29.2)  441 (32.4) | 3,963 (70.8)  920 (67.6) | 0.019 | 2,149 (38.5)  533 (39.2) | 3,437 (61.5)  826 (60.8) | 0.611 |
| **Stroke**  Yes  No | 22 (27.5)  2,051 (29.8) | 58 (72.5)  4,825 (70.2) | 0.651 | 35 (43.8)  2,647 (38.6) | 45 (56.3)  4,218 (61.4) | 0.343 |
| **Ischemic heart disease**  Yes  No | 69 (28.5)  2,004 (29.8) | 173 (71.5)  4,710 (70.2) | 0.655 | 97 (40.1)  2,585 (38.6) | 145 (59.9)  4,118 (61.4) | 0.634 |
| **Retinopathy**  Yes  No | 59 (26.5)  2,014 (29.9) | 164 (73.5)  4,719 (70.1) | 0.267 | 104 (46.6)  2,578 (38.4) | 119 (53.4)  4,114 (61.6) | 0.012 |
| **Nephropathy**  Yes  No | 115 (24.7)  1,958 (30.2) | 351 (75.3)  4,532 (69.8) | 0.012 | 193 (41.4)  2,489 (38.4) | 273 (58.6)  3,990 (61.6) | 0.199 |
| **Foot complications**  Yes  No | 23 (35.4)  2,050 (29.7) | 42 (64.6)  4,841 (70.3) | 0.323 | 29 (44.6)  2,653 (38.6) | 36 (55.4)  4,227 (61.4) | 0.318 |
| **Diabetes treatment modality**  Lifestyle modification only  Oral hypoglycaemic agent (OHA) only  Insulin only  Both OHA and insulin | 57 (32.0)  1,424 (29.9)  150 (31.3)  442 (28.8) | 121 (68.0)  3,340 (70.1)  329 (68.7)  1,093 (71.2) | 0.636 | 58 (32.6)  1,814 (38.1)  187 (39.0)  623 (40.7) | 120 (67.4)  2,943 (61.9)  292 (61.0)  908 (59.3) | 0.111 |
| **Number of antihypertensive agents**  Zero  One  Two  ≥Three | 116 (73.9)  873 (43.0)  785 (30.8)  299 (13.5) | 41 (26.1)  1,157 (57.0)  1,762 (69.2)  1,923 (86.5) | <0.001 | 17 (10.9)  563 (27.8)  916 (36.0)  1,186 (53.4) | 139 (89.1)  1,464 (72.2)  1,627 (64.0)  1,033 (46.6) | <0.001 |
| **Use of lipid-lowering agents**  Yes  No | 1,504 (29.1)  569 (31.7) | 3,658 (70.9)  1,225 (68.3) | 0.040 | 1,983 (38.5)  699 (39.1) | 3,172 (61.5)  1,091 (60.9) | 0.663 |
| **Use of antiplatelet agents**  Yes  No | 700 (30.1)  1,373 (29.7) | 1,628 (69.9)  3,255 (70.3) | 0.730 | 929 (39.9)  1,753 (38.0) | 1,397 (60.1)  2,866 (62.0) | 0.108 |
| **HbA1C, %,** mean ± SD, %  <7.0%  ≥7.0% | 8.07 ± 2.06  784 (27.6)  1,286 (31.3) | 7.83 ± 1.98  2,059 (72.4)  2,821 (68.7) | <0.001  0.001 | 7.88 ± 2.00  1,096 (38.6)  1,585 (38.7) | 7.91 ± 2.00  1,747 (61.4)  2,512 (61.3) | 0.546  0.909 |
| **Systolic BP,** mean ± SD, mmHg  <140 mmHg  140–159 mmHg  160–179 mmHg  ≥180 mmHg | 151.8 ± 13.8  113 (23.1)  1,456 (29.6)  407 (31.8)  97 (35.9) | 150.0 ± 12.9  376 (76.9)  3,460 (70.4)  874 (68.2)  173 (64.1) | <0.001  <0.001 | 151.7 ± 13.6  189 (38.7)  1,796 (36.6)  578 (45.1)  119 (44.1) | 149.9 ± 12.9  300 (61.3)  3,109 (63.4)  703 (54.9)  151 (55.9) | <0.001  <0.001 |
| **Diastolic BP,** mean ± SD, mmHg  <90 mmHg  90–99 mmHg  100–109 mmHg  ≥110 mmHg | 83.6 ± 9.9  1,353 (29.3)  614 (29.9)  92 (38.7)  14 (32.6) | 82.6 ± 9.9  3,270 (70.7)  1,438 (70.1)  146 (61.3)  29 (67.4) | <0.001  0.021 | 82.9 ± 10.1  1,772 (38.4)  789 (38.5)  100 (42.0)  21 (48.8) | 82.9 ± 9.8  2,840 (61.6)  1,263 (61.5)  138 (58.0)  22 (51.2) | 0.939  0.369 |
| **LDL-cholesterol,** mean ± SD, mmol/L  <2.6 mmol/L  ≥2.6 mmol/L | 3.01 ± 1.00  756 (29.6)  1,314 (29.9) | 2.95 ± 0.97  1,800 (70.4)  3,075 (70.1) | 0.042  0.751 | 2.99 ± 0.99  970 (38.1)  1,707 (38.9) | 2.96 ± 0.97  1,578 (61.9)  2,679 (61.1) | 0.174  0.483 |

Abbreviations: AHA, antihypertensive agents; BP, blood pressure; HbA1c, glycosylated haemoglobin A1c; LDL-C, low-density lipoprotein cholesterol; OHA, oral hypoglycaemic agent
