# Supplementary material for: Treatment intensification and therapeutic inertia of antihypertensive therapy among patients with type 2 diabetes and hypertension with uncontrolled blood pressure
Source: Sci Rep. 2024 Jun 1;14:12625. doi: 10.1038/s41598-024-63617-4 (PMC11144228; doi:10.1038/s41598-024-63617-4)
Supplement: Supplementary file 2 — Supplementary Information 2. [file 41598_2024_63617_MOESM2_ESM.docx]

**Supplementary Table S2:** Treatment intensification using different index blood pressure cut-offs and treatments

| Index blood pressure | Total n | Index treatment (Number of AHA) | Treatment intensification | | | |
| --- | --- | --- | --- | --- | --- | --- |
|  |  |  | n | % | 95% Confidence intervals |  |
| ≥140/80 mmHg | 316 | None | 222 | 70.3 | 64.9 – 75.2 |  |
|  | 3,529 | One | 1,298 | 36.8 | 35.2 – 38.4 |  |
|  | 3,728 | Two | 996 | 26.7 | 25.3 – 28.2 |  |
|  | 2,885 | ≥Three | 355 | 12.3 | 11.1 – 13.6 |  |
|  | **10,458** | **Overall** | **2,871** | **27.5** | **26.6 – 28.3** |  |
| ≥140/90 mmHg | 157 | None | 116 | 73.9 | 67.0 – 80.8 |  |
|  | 2,030 | One | 873 | 43.0 | 40.9 – 45.2 |  |
|  | 2,547 | Two | 785 | 30.8 | 29.0 – 32.6 |  |
|  | 2,222 | ≥Three | 299 | 13.5 | 12.0 – 14.9 |  |
|  | **6,956** | **Overall** | **2,073** | **29.8** | **28.7 – 30.9** |  |
| ≥150/95 mmHg | 69 | None | 52 | 75.4 | 65.2 – 85.5 |  |
|  | 847 | One | 431 | 50.9 | 47.5 – 54.3 |  |
|  | 1,286 | Two | 460 | 35.8 | 33.2 – 38.4 |  |
|  | 1,346 | ≥Three | 200 | 14.9 | 13.0 – 16.8 |  |
|  | **3,548** | **Overall** | **1,143** | **32.2** | **30.7 – 33.8** |  |
| ≥160/100 mmHg | 25 | None | 19 | 76.0 | 59.3 – 92.7 |  |
|  | 312 | One | 169 | 54.2 | 48.6 – 59.7 |  |
|  | 590 | Two | 238 | 40.3 | 36.4 – 44.3 |  |
|  | 732 | ≥Three | 118 | 16.1 | 13.5 – 18.8 |  |
|  | **1,659** | **Overall** | **544** | **32.8** | **30.5 – 35.1** |  |
| ≥170/105 mmHg | 7 | None | 6 | 85.7 | 59.8 – 100.0 |  |
|  | 103 | One | 56 | 54.4 | 44.8 – 64.0 |  |
|  | 228 | Two | 107 | 46.9 | 40.5 – 53.4 |  |
|  | 334 | ≥Three | 70 | 21.0 | 16.6 – 25.3 |  |
|  | **672** | **Overall** | **239** | **35.6** | **32.0 – 39.2** |  |
| ≥180/110 mmHg | 2 | None | 2 | 100.0 | - |  |
|  | 37 | One | 22 | 59.5 | 43.6 – 75.3 |  |
|  | 91 | Two | 41 | 45.1 | 34.8 – 55.3 |  |
|  | 161 | ≥Three | 38 | 23.6 | 17.0 – 30.2 |  |
|  | **291** | **Overall** | **103** | **35.4** | **29.9 – 40.9** |  |
