# Supplementary material for: Treatment intensification and therapeutic inertia of antihypertensive therapy among patients with type 2 diabetes and hypertension with uncontrolled blood pressure
Source: Sci Rep. 2024 Jun 1;14:12625. doi: 10.1038/s41598-024-63617-4 (PMC11144228; doi:10.1038/s41598-024-63617-4)
Supplement: Supplementary file 3 — Supplementary Information 3. [file 41598_2024_63617_MOESM3_ESM.docx]

**Supplementary Table S3:** Therapeutic inertia using different index blood pressure cut-offs and treatments

| Index and second blood pressure | Total n | Index treatment (Number of AHA) | Therapeutic inertia | | | |
| --- | --- | --- | --- | --- | --- | --- |
|  |  |  | n | % | 95% Confidence intervals |  |
| ≥140/80 mmHg | 314 | None | 62 | 19.7 | 15.5 – 24.6 |  |
|  | 3,523 | One | 1,534 | 43.5 | 41.9 – 45.2 |  |
|  | 3,721 | Two | 1,880 | 50.5 | 48.9 – 52.1 |  |
|  | 2,881 | ≥Three | 1,819 | 63.1 | 61.4 – 64.9 |  |
|  | **10,439** | **Overall** | **5,295** | **50.7** | **49.8 – 51.7** |  |
| ≥140/90 mmHg | 156 | None | 17 | 10.9 | 6.0 – 15.8 |  |
|  | 2,027 | One | 563 | 27.8 | 25.8 – 29.7 |  |
|  | 2,543 | Two | 916 | 36.0 | 34.2 – 37.9 |  |
|  | 2,219 | ≥Three | 1,186 | 53.5 | 51.4 – 55.5 |  |
|  | **6,945** | **Overall** | **2,682** | **38.6** | **37.5 – 39.8** |  |
| ≥150/95 mmHg | 69 | None | 3 | 4.4 | 0.01 – 12.2 |  |
|  | 846 | One | 124 | 14.7 | 12.3 – 17.0 |  |
|  | 1,284 | Two | 273 | 21.3 | 19.0 – 23.5 |  |
|  | 1,344 | ≥Three | 496 | 36.9 | 34.4 – 39.5 |  |
|  | **3,543** | **Overall** | **896** | **25.3** | **23.9 – 26.7** |  |
| ≥160/100 mmHg | 25 | None | 0 | 0.0 | - |  |
|  | 312 | One | 29 | 9.3 | 6.1 – 12.5 |  |
|  | 590 | Two | 77 | 13.1 | 10.3 – 15.8 |  |
|  | 732 | ≥Three | 165 | 22.5 | 19.5 – 25.6 |  |
|  | **1,659** | **Overall** | **271** | **16.3** | **14.6 – 18.1** |  |
| ≥170/105 mmHg | 7 | None | 0 | 0.0 | - |  |
|  | 103 | One | 6 | 5.8 | 1.3 – 10.4 |  |
|  | 228 | Two | 22 | 9.7 | 5.8 – 13.5 |  |
|  | 334 | ≥Three | 43 | 12.9 | 9.3 – 16.5 |  |
|  | **672** | **Overall** | **71** | **10.6** | **8.2 – 12.9** |  |
| ≥180/110 mmHg | 2 | None | 0 | 0.0 | - |  |
|  | 37 | One | 3 | 8.1 | 0.02 – 21.9 |  |
|  | 91 | Two | 5 | 5.5 | 0.02 – 12.4 |  |
|  | 161 | ≥Three | 11 | 6.8 | 3.5 – 11.9 |  |
|  | **291** | **Overall** | **19** | **6.5** | **3.9 – 10.0** |  |
